# Dolutegravir-induced growth and lifespan effects in *Caenorhabditis elegans*

**DOI:** 10.1186/s40360-023-00715-5

**Published:** 2023-12-07

**Authors:** Shin-Huei Kuo, Wen-Li Hsu, Ching-Ying Wu, Yu-Chang Lai, Tun-Chieh Chen

**Affiliations:** 1 Department of Internal Medicine, Kaohsiung Municipal Ta-Tung Hospital, Kaohsiung Medical University, Kaohsiung, 80145 Taiwan; 2 Division of Infectious Diseases, Department of Internal Medicine, Kaohsiung Medical University Hospital, Kaohsiung Medical University, Kaohsiung, 80708 Taiwan; 3 Department of Dermatology, Kaohsiung Municipal Ta-Tung Hospital, Kaohsiung Medical University Hospital, Kaohsiung Medical University, Kaohsiung, 80145 Taiwan; 4 Regenerative Medicine and Cell Therapy Research Center, Kaohsiung Medical University, Kaohsiung, 80708 Taiwan; 5 Department of Cosmetic Science, Chang Gung University of Science and Technology, Taoyuan, 33303 Taiwan; 6 School of Pharmacy, College of Pharmacy, Kaohsiung Medical University, Kaohsiung, 80708 Taiwan; 7 School of Medicine, College of Medicine, Kaohsiung Medical University, Kaohsiung, 80708 Taiwan; 8 Infection Control Office, Kaohsiung Municipal Ta-Tung Hospital, Kaohsiung Medical University, No. 68, Jhonghua 3rd Rd, Cianjin District, Kaohsiung, 80145 Taiwan; 9 Center for Medical Education and Humanizing Health Professional Education, Kaohsiung Medical University, Kaohsiung, 80708 Taiwan; 10 Center for Tropical Medicine and Infectious Disease Research, Kaohsiung Medical University, Kaohsiung, 80708 Taiwan

**Keywords:** Dolutegravir (DTG), Reactive oxidative species (ROS), People living with HIV (PLWH), Lifespan, *Caenorhabditis Elegans*

## Abstract

**Background:**

Integrase strand transfer inhibitor (INSTIs)-based combination antiretroviral treatment in people living with HIV (PLWH) has been reportedly correlated with several adverse effects, such as weight gain, fetal defects or psychiatric disorders.

**Methods:**

To comprehensively understand the adverse effect of INSTIs, our study utilized *Caenorhabditis Elegans* (*C. elegans*) as a model to investigate how dolutegravir (DTG) affected its life cycle, growth, reproduction and lifespan.

**Results:**

Our results indicated that DTG enhanced body growth at the early stage of treatment, but no change was detected for long-term treatment. The treatment also influenced the reproductive system, decreased egg-hatching but had no effect on egg-laying. Besides, DTG resulted in lifespan reduction, which is dependent on increased levels of reactive oxidative species (ROS) accumulation. Treatment with N-acetyl-cysteine (NAC) in worms restrained intracellular ROS accumulation and improved DTG-induced lifespan reduction.

**Conclusions:**

Our study demonstrates for the first time the effect of DTG treatment on life cycle. DTG-induced adverse effects are potentially associated with intracellular ROS accumulation. Quenching ROS accumulation might provide a novel strategy for dealing with the adverse effects of INSTIs.

## Background

Integrase strand transfer inhibitors (INSTIs) such as dolutegravir (DTG), bictegravir (BIC) and cabotegravir (CAB) are a class of antiretroviral drug for treating most patients who are newly diagnosed with HIV [1]. These drugs reveal the overall good tolerability in clinical practice and characterize a low rate of discontinuation [2]; however, as more HIV patients are treated with INSTIs, recent evidence has revealed weight gain in people living with HIV (PLWH), and up to approximately 30% of the people undergoing this treatment have become overweight [3]. INSTIs, especially DTG at therapeutic doses possesses increased risk for fetal defects in mice studies [4], also affecting mouse neurodevelopment by inhibiting matrix metalloproteinases (MMPs) [5]. For a “return to health” issue in PLWH, there is increasing concern that the adverse effects of INSTIs are becoming disregarded. To maintain health care quality and promote the benefits of PLWH, understanding the adverse effects of INSTIs on PLWH could provide a preventive strategy for treating HIV patients to return to health.

*Caenorhabditis elegans* (*C. elegans*) is a useful model for comprehensive understanding the adverse effect of INSTIs as it possesses uridine diphosphate (UDP)-glucuronosyltransferase 25 (*ugt-25*) and cytochrome P450 13A7 (*cyp-13A7*) respectively belonging to the homologous genes of human *UGT1A1* and *CYP3A4* able to metabolize DTG and BIC [6, 7]. Besides, *C. elegans* also has a simple anatomy, a short lifespan and the capability to self-reproductive; these characteristics support a powerful experimental system to firstly clarify the effect of drug on the whole life cycle.

Accordingly, this study utilized *C. elegans* as a model to investigate how DTG affected the worms’ life cycle. Worm growth, reproduction and lifespan were examined with DTG treatment from early adulthood to death. It was also demonstrated that an increased level of intracellular reactive oxidative species (ROS) production was involved in DTG-induced adverse effects. These results explored the potential adverse effects of DTG on ontogenesis in PLWH.

## Methods

### *C. Elegans* culture and DTG treating protocol

*C. elegans* (Bristol strain N2) was attained from the Caenorhabditis Genetics Center (CGC), cultivated on nematode growth medium (NGM) agar plates, and fed with UV-killed *Escherichia coli* (*E. coli*) OP50 to prevent the potential confounding effects of bacterial metabolism. Then, the worms were dissolved in alkaline bleach solution to collect eggs that were hatched on the NGM plates at 20℃ for 48 h while provided with food to obtain age-synchronized populations. To mimic the patient’s receipt of DTG (ViiV Healthcare, UK), synchronized early adulthood (Day 0) worms were seeded onto NGM plates and fed with UV-killed *E. coli* treated in different doses of DTG until death. Four nominal DTG doses, which were referred to in previous studies [5], were used in this study, namely 0 µg/ml (control), 0.83 µg/ml, 8.3 µg/ml, and 83 µg/ml, and combination of treatment with 5 mM N-acetyl-cysteine (NAC, Sigma-Aldrich, USA) for further lifespan assay and intracellular ROS production analysis [8]. Because DTG was dissolved in dimethyl sulfoxide (DMSO, Sigma-Aldrich), the final DMSO concentration in control group was 0.4%. The body size of worms was detected using a NIS-Elements AR Object Tracking module at the indicated time (NIKON, Japan).

### Egg-laying and egg-hatching assays

After treating at different doses of DTG for two days or four days, one worm was transferred to each of the wells of a 12-well NGM plate with a bacteria lawn for the egg-laying and egg-hatching assays as previously described [9]. Briefly, each worm was transferred to a new plate within the duration of the egg-laying period. The total number of eggs for each of the worms was recorded for egg-laying assay in which the old plates containing the eggs were then hatched and incubated to L4 worms for easier counting of progeny. Twelve worms were evaluated for each of the treatment doses.

### Lifespan assay

The tested worms were moved to new 60 mm agar dishes that were seeded with *E. coli OP50*, and marked as lifespan assay day 0 when growing to the L4 stage. Besides, the worms were transferred to new agar dishes daily during the first 4–5 days to avoid mixing generations. The worms that showed no response to gentle prodding with a platinum wire and no pharyngeal pumping were considered to have expired. Three biological replicates were performed, and a total of 120 worms were assayed [10].

### Analysis of intracellular ROS production

The ROS accumulation levels of worms were detected by dihydroethidium (DHE, Sigma-Aldrich) and Mito-SOX (Molecular Probes, Thermo Fisher Scientific, USA) staining. Briefly, approximately 100 worms were transferred to microtubes containing 5 µM DHE or 5 µM Mito-SOX in K-medium and incubated for 30 min at 25 ℃. After staining, worms were washed by PBS and fixed by 10% formaldehyde (Sigma-Aldrich) for 30 min. The intracellular ROS production in worms was observed with an upright fluorescence Microscope (AxioPlan 2, Zeiss, Germany) or an Olympus Cell^R IX81 fluorescence microscope (Olympus, Japan).

### Statistical analysis

GraphPad Prism version 5.0 (Dotmatics) was utilized to create bar charts; error bars pointed out standard deviations unless otherwise noted. One-way ANOVA followed by Bonferroni’s post-hoc test and two-tailed, paired or unpaired Student’s *t*-tests were used to compare the differences between groups. In addition, survivorship analysis was performed using the Statistical Product and Service Solutions (SPSS, version 12.0, USA). A *p*-value of less than 0.05 for the difference between groups was considered statistically significant.

## Results

### Effect of DTG treatment on body growth in worm

**Fig. 1:**
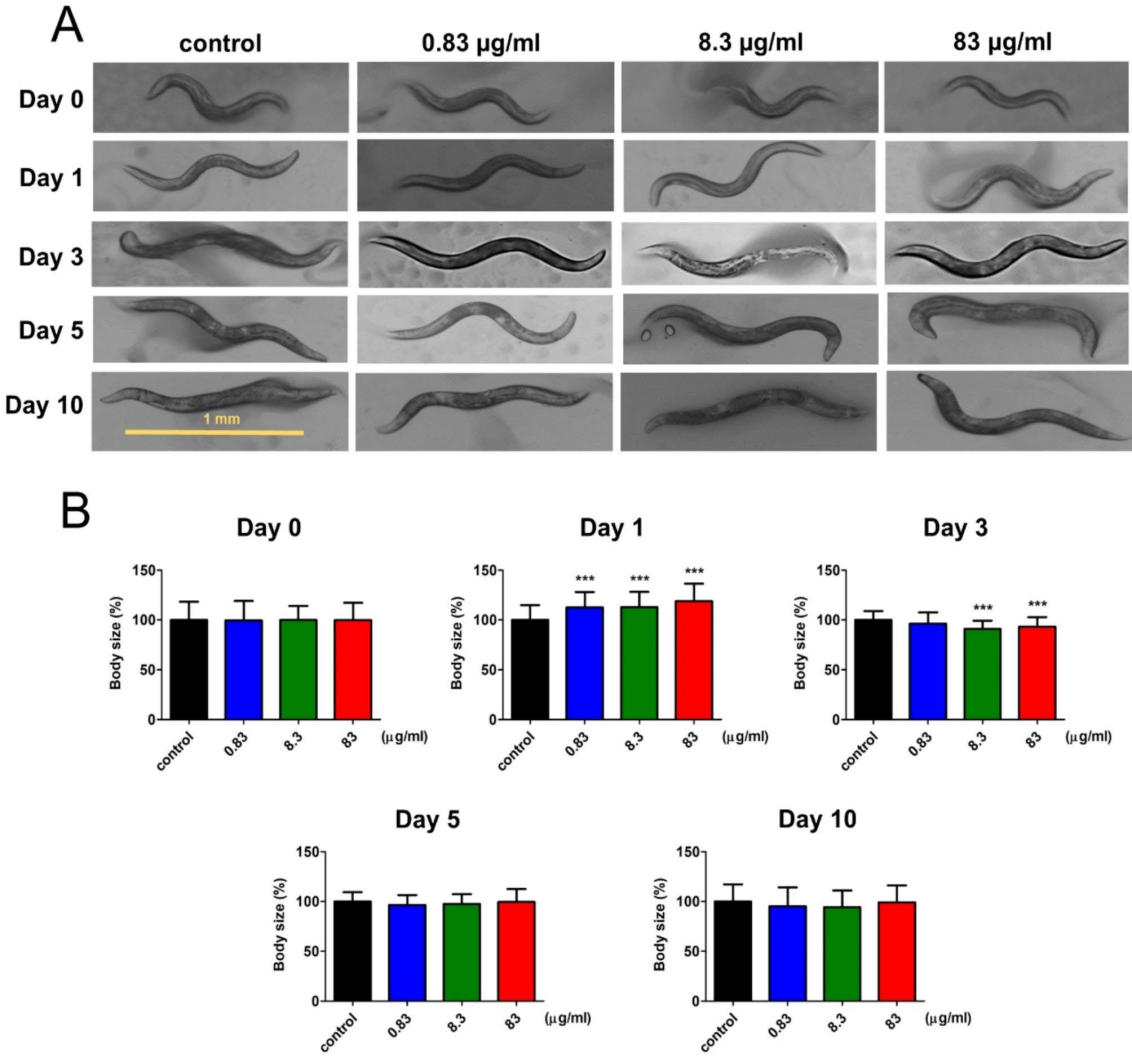
Body size evaluation in worms with DTG treatment. (**A**) After treatment at different concentrations of DTG from Day 0 (early adulthood) to Day 10, worm size was observed at the indicated times. Quantification of body size as (**B**). The data shown represent the average of three independent experiments, and each experiment measured for 120 worms (mean ± SD, ***p < 0.001)

A previous study indicated that DTG-associated weight gain is due to inhibition of the melanocortin system (MCS) that plays a crucial role in controlling appetite and metabolic energy balance [11], where interrupting MCS by DTG facilitates increase in appetite and cause of weight gain. Because *C. elegans* also possesses a cluster of genes similar to MCS functions [12–14], the body growth was observed at the indicated time with different doses of DTG treatment. As shown in Fig. 1A, worm body size was a mere increase at Day 1 compared to the control group, but there was no difference between each group from Day 2 to Day 10; quantifying body size at the indicated time revealed a significant increase after treating with DTG for 1 day (Fig. 1B), so DTG only influenced body growth at the early stage of treatment thereby potentiating no change for long-term DTG treatment.

### Effect of DTG treatment on the reproductive system in worm

**Fig. 2:**
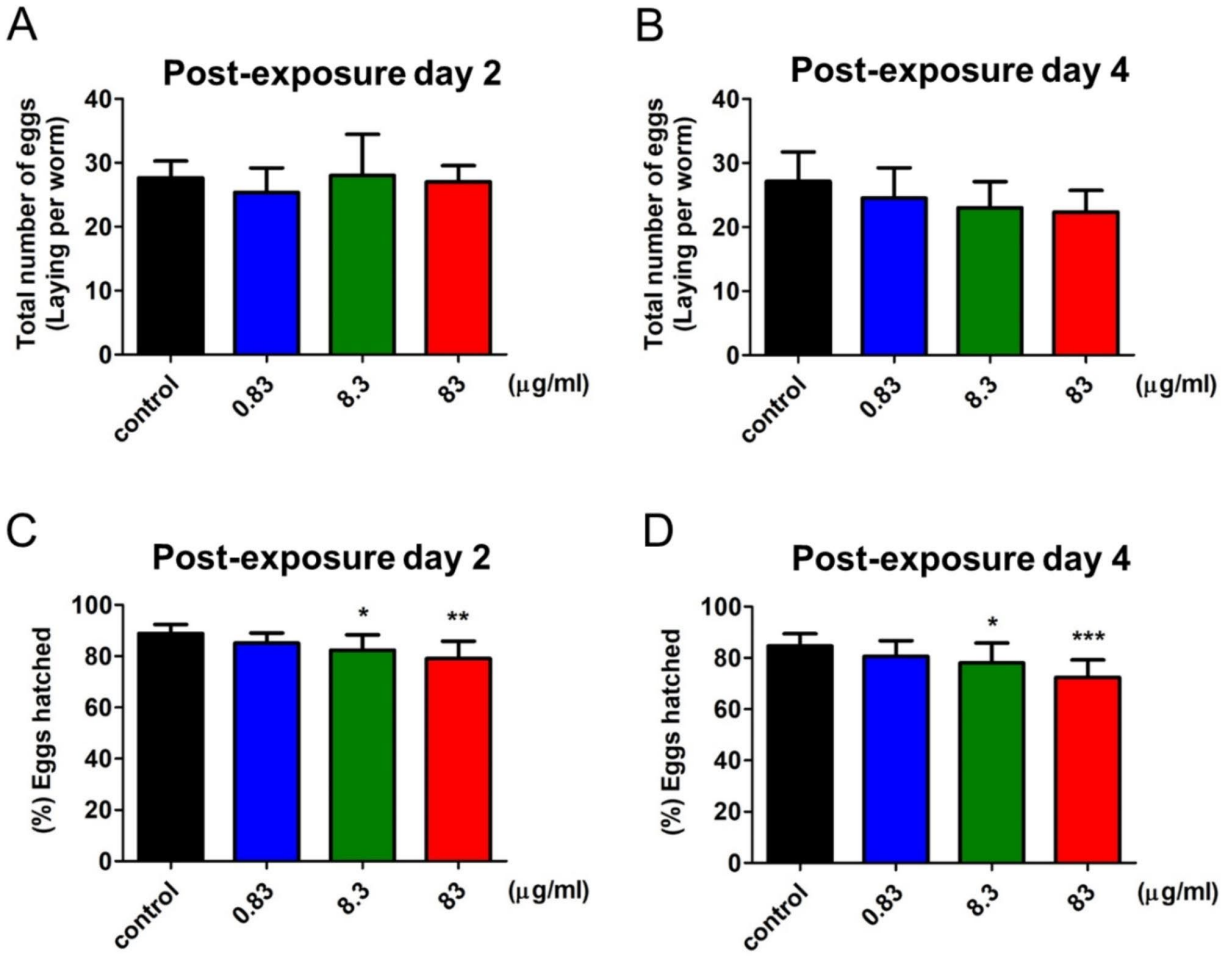
Effect of DTG on *C. elegans* egg-laying and egg-hatching. The number of eggs laid was observed in worms that received DTG treatment for (**A**) two days and (**B**) four days. Percentage of eggs that hatched from the gravid worms treated with DTG for (**C**) two days and (**D**) four days (mean ± SD, *p < 0.05; **p < 0.01; ***p < 0.001)

Current studies have shown that using the self-progeny of *C. elegans* is an effective parameter utilized to determine the extent of reproductive disability [9, 15, 16]. Previous studies mentioned DTG at therapeutic doses held increased risk for fetal defects in mice models; we next detected whether DTG treatment in worms also affected the reproductive system. The total number of eggs laid and the percentage of eggs hatching were determined after treatment with DTG for two days or four days within the duration of the egg-laying period. Our result found no significant effect of DTG on the total number of eggs laid after exposure to DTG for two days or four days, although DTG dose-dependently reduced the total number of eggs laid at post-exposure day 4 (Fig. 2A and B). Nevertheless, the egg-hatching was significantly decreased by DTG, revealed in a dose-dependent manner (Fig. 2C and D). A potential reproductive disability was therefore observed in *C. elegans* with DTG treatment, especially inhibition of egg-hatching, demonstrating that DTG attenuated egg-hatching but did not affect egg-laying.

### DTG attenuates worms’ lifespan

**Fig. 3:**
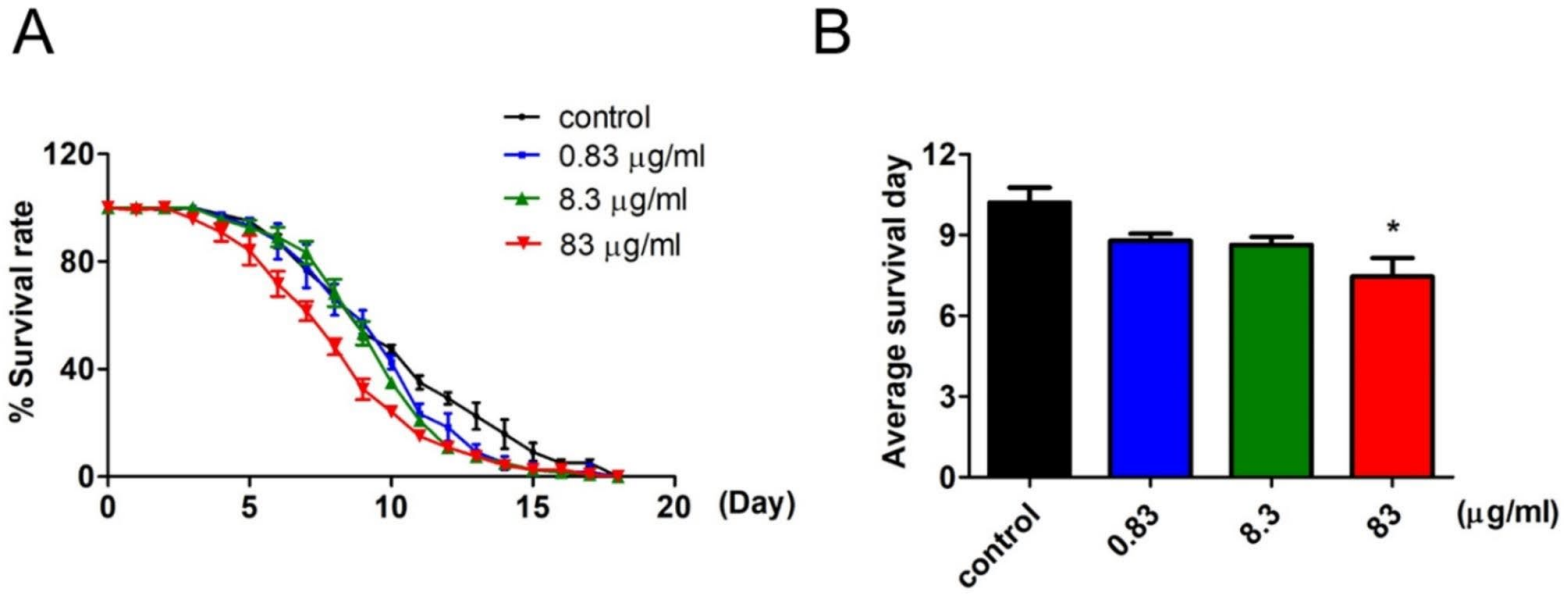
Effect of DTG on lifespan evaluation in *C. elegans*. Worms were treated at different concentrations of DTG from early adulthood to death. (**A**) Kaplan–Meier curves with univariate analyses for worms (n = 120). (**B**) Average survival days of three independent experiments were analyzed by (**A**). The data are presented as compared to the control group (mean ± SD, *p < 0.05)

DTG in clinical performance displays overall good tolerability and low rate of discontinuation [2], implying that PLWH could receive DTG treatment long-term to return to health. To mimic the effect of long-term DTG treatment on lifespan, worms were treated with different doses of DTG from early adulthood to death, and then their survival days were observed and counted. The results indicated that 83 µg/ml of DTG significantly reduced *C. elegans* lifespan (Fig. 3A and B). Although treatment with 0.83 and 8.3 µg/ml of DTG did not significantly affect the worm’s average survival day, the curves of survival rate dramatically dropped after Day 10 (Fig. 3A and B). Day 10 for the worms is approximately middle adulthood in mice where half of gene mutations occurs through the natural process of aging; half of gene mutations contributes to genomic instability, promoting aging and age-associated diseases with intracellular ROS accumulation [17, 18]. Consequently, treatment with DTG might cause intracellular ROS accumulation and thereby inhibit the worms’ lifespan.

### DTG treatment enhances intracellular ROS accumulation in worm

**Fig. 4:**
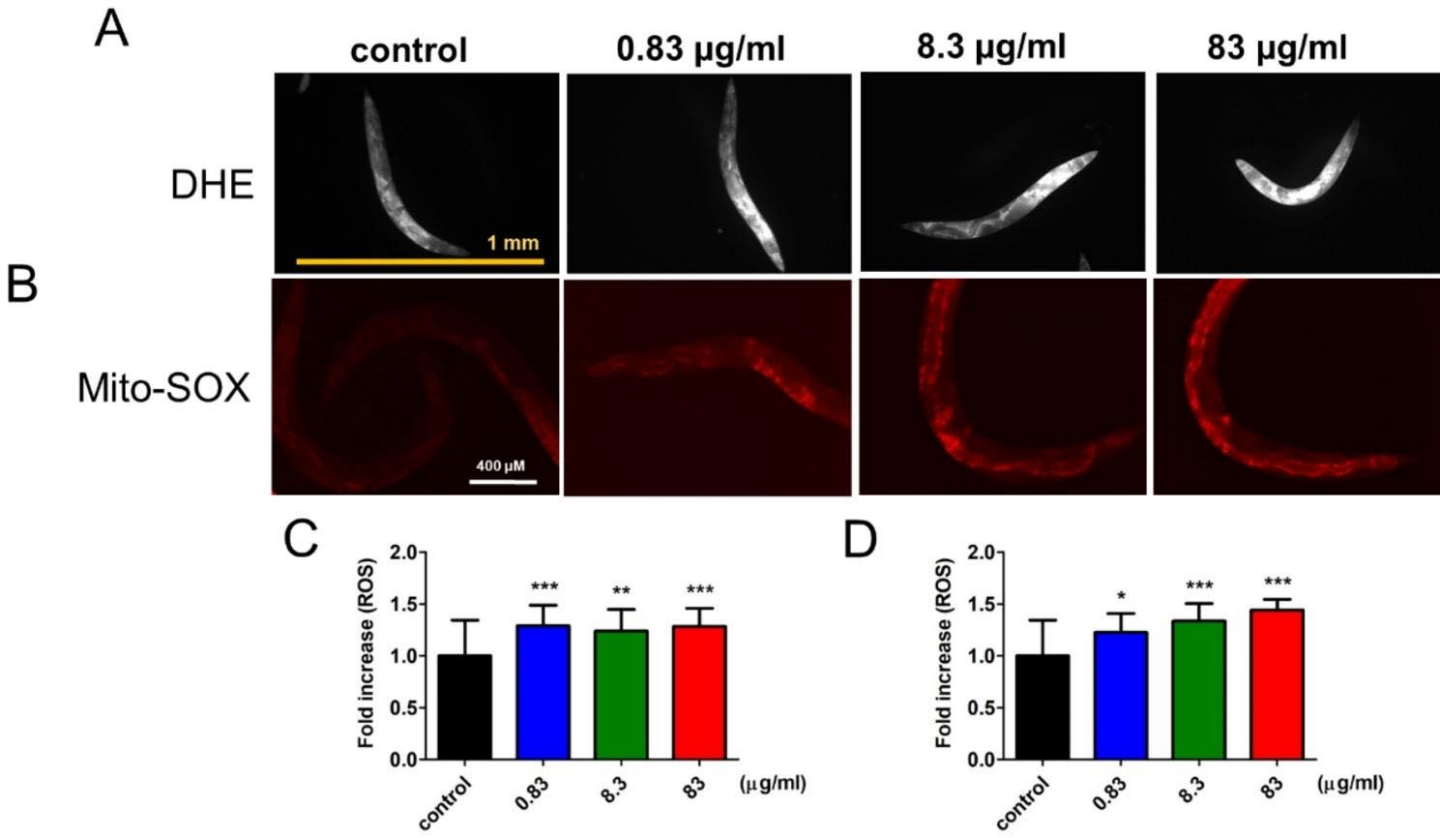
Effect of DTG on intracellular ROS accumulation level in worms. Worms were treated by DTG from early adulthood (Day 0) to Day 10 at different concentrations, and intracellular ROS levels were analyzed on the 10th day by staining with (**A**) DHE and (**B**) Mito-SOX. The intensity of emitted fluorescence was quantified as (**C**) and (**D**) respectively. The data shown represent the average of four independent experiments, and each experiment measured for 84 worms (mean ± SD, *p < 0.05; **p < 0.01; ***p < 0.001)

We validated whether DTG-induced lifespan decrease was due to intracellular ROS accumulation. Because treatment with DTG influenced the curve of survival rate after Day 10, worms were stained with intracellular ROS indicators, DHE and Mito-SOX at Day 10. As shown in Fig. 4A, the levels of intracellular ROS production potentiates upregulation with DTG treatment at Day 10, and the quantification of fluorescence intensity showed significant increase (Fig. 4B). Based on the oxidative stress theory of aging, ROS are continuously produced in mitochondria because stepwise reduction of O_2_ produces several ROS within energy production [19]. To confirm that DTG treatment-induced intracellular ROS accumulation was from mitochondria, we utilized Mito-SOX indicator, which is a modified DHE analog derived by the addition of a triphenyl-phosphonium group, which can specifically target mitochondria ROS production [20]. Our findings showed a dose-dependent increase in mitochondria ROS accumulation with DTG treatment (Fig. 4C and D), implying that treatment with DTG in worms potentiates promotion of the aging process and influence on worms’ lifespan. These results indicated that intracellular ROS accumulation was involved in regulating DTG-induced lifespan attenuation.

### Treatment with NAC in worms restrains intracellular ROS accumulation and extends DTG-induced lifespan reduction

**Fig. 5:**
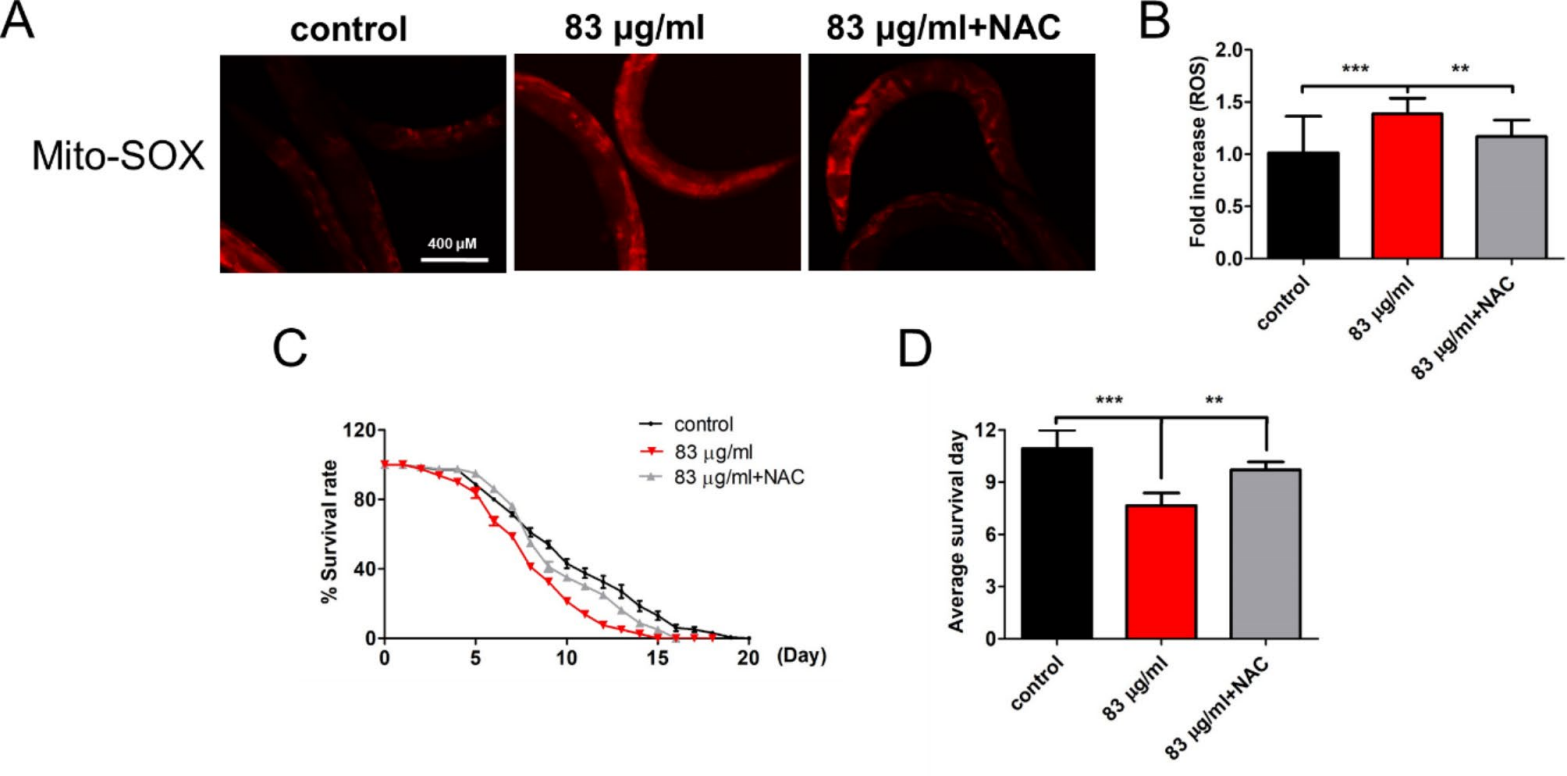
Effect of NAC treatment on lifespan and ROS accumulation of nematodes exposed to 83 µg/ml of DTG. (**A**) After co-treatment with 83 µg/ml of DTG and 5mM NAC, intracellular ROS levels were analyzed on the 10th day by staining with Mito-SOX and quantified as (**B**). (**C**) Kaplan–Meier curves with univariate analyses for worms (n = 120). (**D**) Average survival days of three independent experiments were analyzed by (**C**) (mean ± SD, **p < 0.01; ***p < 0.001)

To further demonstrate the effect of ROS accumulation on DTG-induced lifespan reduction in worms, an antioxidant NAC was utilized to quench DTG-induced mitochondria ROS accumulation [8]. 83 µg/ml of DTG combined treatment with 5mM NAC, the levels of intracellular ROS accumulation by DTG treatment were restrained by NAC at Day 10 (Fig. 5A and B). Our findings also suggested that DTG-induced *C. elegans* lifespan decrease was significantly extended by NAC, and a short average survival day with DTG induction was rescued by NAC (Fig. 5C and D). Our study illustrates that DTG causes lifespan attenuation related to intracellular ROS accumulation. Quenching DTG-induced ROS accumulation by NAC has significantly increased worms’ lifespan.

## Discussion

Our study used *C. elegans* as a model to identify the effect of DTG on life cycle. We found that DTG promoted worms’ body growth at the early stage of treatment, but no change was found for long-term treatment. DTG treatment in worms also influenced the reproductive system, reduced the egg-hatching although had no effect on egg-laying but did result in lifespan reduction, which is dependent on increased levels of ROS accumulation; thus, treatment with NAC in worms to quenching DTG-induced ROS accumulation rescued lifespan reduction. According to previous research, enhancement of body growth potentiates induction of senescence because of huge oxidative damage accumulation [21]. Despite worms’ body size revealing no change for long-term DTG treatment, DTG at the early stage of treatment not only increased the body size but also decreased the lifespan. Similarly, clinical data reveals no overall change in the rate of weight gain after treatment with INSTIs in PLWH [22]. INSTIs-associated weight gain perhaps occurs at the early stage of treatment, but there is no long-term cohort study concerning INSTIs treatment to date.

The mechanism of DTG-induced worm body size increase could be due to inhibition of MCS. Melanocortin 4 receptor (MC4R), one of the most important receptors in MCS at second-order neurons to control appetite and metabolic energy balance [23]. The MC4R receptor could be targeted by DTG, contributing to appetite increase and weight gain in PLWH with interference on anorexigenic signal [11]. Even though there is no ortholog of MC4R receptor in *C. elegans*, a cluster of genes, *dop-1*, *dop-3*, *dop-4* and *ser-4*, which belong to the G protein-coupled receptor (GPCR) as food sensors, functionally similar to MC4R receptor can regulate foraging and food intake when animals encounter food [12, 13]. Our future work will investigate the effect of DTG on *dop-1*, *dop-3*, *dop-4* and *ser-4*, clarifying the underlying mechanism of DOT-induced worm body size increase. It might also explain the mechanism of INSTIs-associated weight gain in PLWH.

Our results showed DTG-induced reproductive toxicity in decrease in worm’s egg-hatching. This result was similar to previous research where DTG at therapeutic doses possessed increased risk for fetal defects in mice models [4]. Actually, DTG has the potential to affect prenatal and postnatal neurodevelopment via blocking matrix MMPs activities during gestation [5]. In *C. elegans*, neurotoxicity can be induced by ROS accumulation and disturbance of neuronal activity [24]. It could be that DTG-induced ROS accumulation interrupts egg-hatching through blocking neuronal activity. Interestingly, a retrospective cohort study revealed that the percentage of (human) infants with adverse outcomes is no different when pregnant women are exposed to INSTI and non-INSTI antiretroviral therapy [25]; there is also no cluster of birth defect type and no neural tube defects are observed [26]. Nevertheless,, switching antiretroviral therapy to DTG in PLWH has a risk of psychiatric disorders, which are related to oxidative stress in neurons [27, 28]. The effect of DTG on neuronal activity and neurobehavioral development requires validation in future studies.

## Conclusions

Overall, our study demonstrates for the first time the effect of DTG treatment on life cycle. To return to health, PLWH have to receive INSTIs for long-term, but INSTIs, especially DTG might provide a risk of lifespan attenuation due to intracellular ROS accumulation based on our *C. elegans* study. INSTIs-associated weight gain and becoming overweight illustrate PLWH have potential risk for developing age-associated morbidities [29]. Therefore, quenching ROS accumulation by health education models in PLWH such as exercise, food administration or nutritional supplements intake could provide a novel strategy for dealing with the adverse effects of INSTIs.

## Data Availability

All data generated or analyzed during this study are included in this published article. The datasets used and/or analyzed during the current study are available from the corresponding author on reasonable request.
